# Role of Ccr4-Not complex in heterochromatin formation at meiotic genes and subtelomeres in fission yeast

**DOI:** 10.1186/s13072-015-0018-4

**Published:** 2015-08-15

**Authors:** Cristina Cotobal, María Rodríguez-López, Caia Duncan, Ayesha Hasan, Akira Yamashita, Masayuki Yamamoto, Jürg Bähler, Juan Mata

**Affiliations:** Department of Biochemistry, University of Cambridge, Cambridge, UK; Department of Genetics, Evolution and Environment, UCL Cancer Institute, University College London, London, UK; Laboratory of Cell Responses, National Institute for Basic Biology, Okazaki, Japan

**Keywords:** Ccr4-Not complex, Genome-wide approaches, RIP-chip, ChIP-seq, Heterochromatin, *S. pombe*

## Abstract

**Background:**

Heterochromatin is essential for chromosome segregation, gene silencing and genome integrity. The fission yeast *Schizosaccharomyces pombe* contains heterochromatin at centromeres, subtelomeres, and mating type genes, as well as at small islands of meiotic genes dispersed across the genome. This heterochromatin is generated by partially redundant mechanisms, including the production of small interfering RNAs (siRNAs) that are incorporated into the RITS protein complex (RNAi-Induced Transcriptional Silencing). The assembly of heterochromatin islands requires the function of the RNA-binding protein Mmi1, which recruits RITS to its mRNA targets and to heterochromatin islands. In addition, Mmi1 directs its targets to an exosome-dependent RNA elimination pathway.

**Results:**

Ccr4-Not is a conserved multiprotein complex that regulates gene expression at multiple levels, including RNA degradation and translation. We show here that Ccr4-Not is recruited by Mmi1 to its RNA targets. Surprisingly, Ccr4 and Caf1 (the mRNA deadenylase catalytic subunits of the Ccr4-Not complex) are not necessary for the degradation or translation of Mmi1 RNA targets, but are essential for heterochromatin integrity at Mmi1-dependent islands and, independently of Mmi1, at subtelomeric regions. Both roles require the deadenylase activity of Ccr4 and the Mot2/Not4 protein, a ubiquitin ligase that is also part of the complex. Genetic evidence shows that Ccr4-mediated silencing is essential for normal cell growth, indicating that this novel regulation is physiologically relevant. Moreover, Ccr4 interacts with components of the RITS complex in a Mmi1-independent manner.

**Conclusions:**

Taken together, our results demonstrate that the Ccr4-Not complex is required for heterochromatin integrity in both Mmi1-dependent and Mmi1-independent pathways.

**Electronic supplementary material:**

The online version of this article (doi:10.1186/s13072-015-0018-4) contains supplementary material, which is available to authorized users.

## Background

Carbon catabolite repression 4-negative on TATA-less (Ccr4-Not) is a highly conserved multiprotein complex that regulates gene expression at multiple levels, including transcription initiation and elongation, RNA export, RNA turnover and translation [1]. The Ccr4-Not complex in *Saccharomyces cerevisiae* consists of nine core subunits (Ccr4, Caf1/Pop2, Not1-5, Caf40 and Caf130), most of which are present in other eukaryotic organisms [1]. Two enzymatic activities are associated with the complex: deadenylation (carried out by Ccr4 and Caf1/Pop2, which are unrelated to each other in sequence) and ubiquitination (performed by Not4, which is also known as Mot2) [1]. The control of cytoplasmic mRNA turnover by Ccr4-Not is the best understood function of the complex. This role is mediated by Ccr4-Not deadenylase activity, which reduces the length of the poly(A) tail that protects mRNAs from degradation and thus destabilizes them [1]. Ccr4-Not is also involved in nuclear RNA degradation, and associates with the nuclear exosome and the non-canonical polyadenylation complex TRAMP (Trf4/Air2/Mtr4 Polyadenylation), which recognizes and tags aberrant mRNAs [2]. Ccr4-Not can also regulate mRNA translation independently of its deadenylase activity [3, 4]. Other roles of the complex such as the regulation of transcription and RNA export are less understood, but do not appear to be connected with specific enzymatic activities [1]. Some of these functions may involve interactions between Ccr4-Not and histone acetyl transferases [5] or RNA polymerase II [6]. Finally, the Not4 subunit possesses E3 ubiquitin ligase activity and is involved in protein quality control [7]. Ccr4-Not is recruited to target mRNAs by sequence-specific RNA-binding proteins that interact with different subunits of the complex [8–11]. In mammalian cells, the Ccr4-Not complex is also recruited to microRNA (miRNA) targets through interactions with the GW182 protein, which in turn is directed to miRNA binding sites by argonaute proteins [12–14].

The fission yeast *Schizosaccharomyces pombe* is widely used as a model for the regulation of chromatin and gene expression in eukaryotic cells. The presence of a core RNAi machinery (including argonaute (Ago1), Dicer (Dcr1) and RNA-dependent RNA polymerase (Rdp1), which are absent in *S. cerevisiae*) makes it a particularly attractive system [15]. *S. pombe* contains heterochromatin at three main sites: pericentromeres, subtelomeres and mating type region [16]. In addition, there are short blocks of heterochromatin at a small number of loci spread throughout the genome (heterochromatin islands) [17], and additional heterochromatin domains (HOODs) appear when the nuclear exosome is inactivated [18]. RNAi mediates the formation of heterochromatin at the major sites. Transcription from repeated elements within these regions causes the formation of double stranded RNAs (dsRNAs), which are processed by Dcr1 to produce small interfering RNAs of 21–22 nucleotides (siRNAs) that are loaded onto the RNA-induced transcriptional silencing (RITS) complex. RITS is composed of Ago1, the chromodomain protein Chp1 and a structural protein called Tas3. RITS is targeted by the siRNAs to the sequence repeats, where it recruits the Clr4 protein that catalyses the methylation of histone H3 in lysine 9 (H3K9), a hallmark of heterochromatin. Chp1 binds to methylated H3 (H3K9-me), thus generating a positive feedback that promotes the assembly and spreading of heterochromatin. Heterochromatin in these regions can also be assembled by a less well-characterized, RNAi-independent pathway [16]. HP1 protein family members (such as Swi6) are recruited to methylated H3K9 and form a platform that recruits other chromatin modifiers, allowing the spread of heterochromatin [16]. Other *cis* sequences and *trans* factors act as boundary elements, preventing heterochromatin from spreading into adjacent regions. A key factor is a protein called Epe1, which is recruited to heterochromatin and acts as an anti-silencing element [19–23].

*S. pombe* cells enter sexual differentiation, a process that culminates in meiosis and sporulation, under nitrogen starvation conditions [24]. This developmental process is accompanied by a complex gene expression program [25], involving both transcriptional and posttranscriptional mechanisms [25–32]. The expression of some meiotic genes in mitotically dividing cells (mostly belonging to the ‘early’ category) is toxic, and is prevented by a silencing mechanism mediated by the YTH (YT521-B Homology) family RNA-binding protein Mmi1 [27]. Mmi1 recognizes a specific sequence on its target RNAs (known as Determinant of Selective Removal, or DSR) and tags them for degradation by the nuclear exosome [27, 33]. This pathway involves the addition of a poly(A) tail to Mmi1-bound RNAs, and requires the function of nuclear poly(A) binding protein (Pab2) [33–35] as well as the zinc-finger-containing protein Red1 [34, 36].

Several heterochromatin islands overlap with Mmi1 target genes [17]. These loci associate with Ago1 [17], Rrp6 and Red1 [37, 38], and some of them lose H3K9 methylation in the absence of Red1 [37, 38] and Mmi1 [37, 39]. By contrast, other islands that do not correspond to meiotic genes fail to accumulate Red1 and are not sensitive to *red1* mutation [38]. Insertion of a DSR sequence is sufficient to induce the formation of a heterochromatin region [37, 38], although additional *cis* sequences are likely to be important [39]. This process requires active transcription as well as *mmi1*, *red1* and *rrp6* functions [37–39]. RITS components interact with heterochromatin islands in a Mmi1-dependent manner [37, 38], although *ago1* and *dcr1* are not necessary for their integrity. In addition, RITS associates with Mmi1 target mRNAs, in a process that also requires Mmi1 [37]. Pab2 has a major influence in the recruitment of RITS to *mei4* RNA but a smaller importance for its binding to the *mei4* gene, while Red1 has the opposite effect. This finding suggests the existence of parallel, partially redundant pathways to recruit RITS to heterochromatin islands [37]. In contrast to heterochromatin islands, H3K9 methylation in HOODs is dependent on RNAi [18].

We show here that Mmi1 recruits the conserved Ccr4-Not complex to its RNA targets. Mutations in genes encoding subunits of the complex (*ccr4*, *caf1*) do not affect the stability of Mmi1 RNA targets, but lead to the loss of heterochromatin in the corresponding loci. Moreover, *ccr4* and *caf1* mutations disrupt heterochromatin in subtelomeric regions, which are not affected by *mmi1* mutations. The deadenylase activity of Ccr4 is essential for both functions. Two other subunits of the complex show specific behaviours: Mot2/Not4 is required at both islands and subtelomeres, whereas Not2 only has an effect on heterochromatin islands. Finally, we show that the Ccr4-Not complex associates with the RITS complex in a Mmi1-independent fashion. Our results demonstrate that the Ccr4-Not complex is required for heterochromatin integrity in both Mmi1-dependent and Mmi1-independent manners.

## Results

### The Mmi1 protein recruits the Ccr4-Not complex to its target mRNAs

**Fig. 1:**
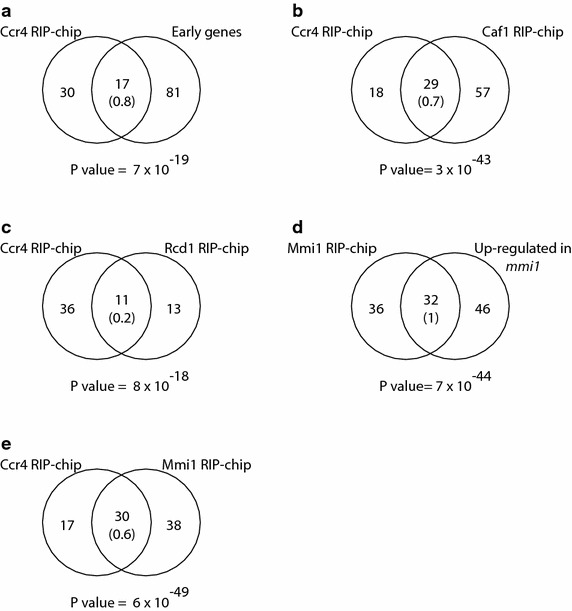
Ccr4-Not associates with Mmi1 RNA targets. Venn diagrams comparing RIP-chip and microarray-based gene expression experiments. The *numbers* in *parentheses* indicate the expected overlap if randomly generated lists of the corresponding sizes were used. The *numbers* below the diagrams show the p value of the observed overlap (see “Methods”). **a** mRNAs associated with Ccr4 and early meiotic genes. **b** mRNAs associated with Ccr4 and with Caf1. **c** mRNAs associated with Ccr4 and with Rcd1. **d** mRNAs associated with Mmi1 and genes overexpressed in *mmi1Δ mei4Δ* mutants. **e** mRNAs associated with Ccr4 and with Mmi1.

As part of a project to understand the regulation of RNA decay in fission yeast, we sought to identify mRNAs associated with the Ccr4-Not complex in vegetatively growing cells. We purified epitope-tagged Ccr4 together with interacting RNAs, and used DNA microarrays to identify the bound RNAs (RNA-binding protein immuno precipitation analysed with DNA chips, or RIP-chip). Ccr4 copurified with ~40 mRNAs and five non-coding RNAs (ncRNAs) that were highly enriched in early meiotic genes (Fig. 1a; Additional file 1: Table S1); these genes are weakly expressed in vegetative cells but are induced during pre-meiotic S phase and meiotic prophase [25]. Two other subunits of the complex, Caf1 and Rcd1 (the *S. pombe* Caf40 homologue), interacted with the same set of RNAs, suggesting that the whole Ccr4-Not complex associates with these mRNAs (Fig. 1b, c; Additional file 1: Table S1). Although the overlap between mRNAs associated with different subunits was highly significant, it was far from complete. This may be due to technical noise (some interactions may be lost during the purification) or reflect the existence of multiple complexes containing different subunits. At present, we cannot distinguish between these two possibilities.

A subset of early meiotic genes binds to the YTH-domain-containing Mmi1 protein, which targets them for degradation via the nuclear exosome pathway. Although several targets of Mmi1 have been identified by Mmi1 immunoprecipitation coupled with RT-PCR [37], they have not been analysed systematically. To obtain a full set of direct Mmi1 targets, we performed RIP-chip experiments using epitope-tagged Mmi1, complemented with microarray-based expression profiling of *mmi1* deletion mutants. As *mmi1* is an essential gene, the expression arrays were performed in a *mei4* deletion background (*mei4* encodes a meiotic transcription factor), which suppresses the lethality of the *mmi1* deletion [27]. The Mmi1 protein coprecipitated with 61 mRNAs and 7 ncRNAs (Additional file 1: Table S1), whereas 69 mRNAs and 9 ncRNAs were overexpressed in *mmi1* mutants (Additional file 1: Table S2). Both sets overlapped extensively with each other (Fig. 1d), as well as with genes overexpressed in mutants in the *pab2* and *red1* genes [34]. The 68 Mmi1-associated RNAs encompassed 17 out of the 21 previously published Mmi1 targets [37]. Direct targets of Mmi1 overlapped with Ccr4-bound mRNAs, indicating that both proteins bound to the same RNA set (Fig. 1e). Two annotated ncRNAs coprecipitated with Ccr4, Caf1, Rcd1 and Mmi1: *meiRNA* (*SPNCRNA.103*), which is a key regulator of entry into pre-meiotic S phase [40] and a known Mmi1 interactor [27], and the uncharacterised *SPNCRNA.388*. We note that *SPNCRNA.388* contains a small translated open reading frame, indicating that it has coding potential [41].

**Fig. 2:**
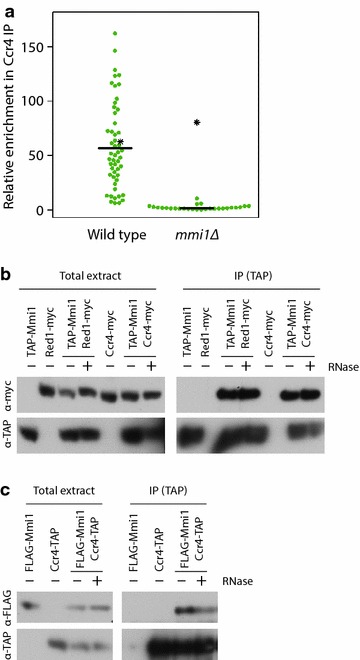
Mmi1 recruits Ccr4-Not to its mRNA targets. **a** Relative enrichment of Mmi1 targets in Ccr4 RIP-chip experiments (normalized to actin) in wild-type cells (*left*) and *mmi1* mutants. Each *dot* corresponds to an individual mRNA, and the *not1* mRNA is designated with a *star*. *Horizontal lines* the median enrichment of all Mmi1 targets. **b** Cell extracts were prepared from the indicated strains and mock-treated (−) or treated with RNase (+). Immunoprecipitations were carried out using antibodies against TAP. Samples were probed with antibodies against myc (*top*) or TAP (*bottom*). The interaction between TAP-Mmi1 and Red1-myc was used as a positive control. **c** As in B, but samples were probed with antibodies against a FLAG epitope (*top*) or TAP (*bottom*). Similar results were obtained with different epitope tag combinations of Mmi1 and Ccr4.

Mmi1 is thought to bind directly to a well-defined RNA sequence motif [42], while the Ccr4-Not complex is typically recruited to particular mRNAs through sequence-specific RNA-binding proteins [1, 43]. This result raised the possibility that Ccr4-Not is recruited to its RNA targets by Mmi1. To investigate this possibility further, we performed Ccr4 RIP-chip experiments in cells lacking *mmi1* (as above, the experiment was performed in a *mei4Δ* background). As predicted, the interaction between Ccr4 and its RNA targets was completely lost in the absence of Mmi1 (Fig. 2a). By contrast, the mRNA encoding Not1, another component of the Ccr4-Not complex, was enriched in both wild-type and *mmi1* mutant backgrounds (Fig. 2a, indicated with a star). The interaction between Ccr4 and the *not1* mRNA is likely to represent a cotranslational interaction between Ccr4 and the Not1 nascent peptide [44, 45], and serves as a control for the immunoprecipitation reaction. In addition, the Mmi1 and Ccr4 proteins coprecipitated in an RNA-independent manner, indicating that they are part of the same multiprotein complex (Fig. 2b, c). Taken together, these data indicate that Mmi1 recruits the Ccr4-Not complex to its target mRNAs.

### Ccr4-Not does not regulate the stability and translation of Mmi1 targets

**Fig. 3:**
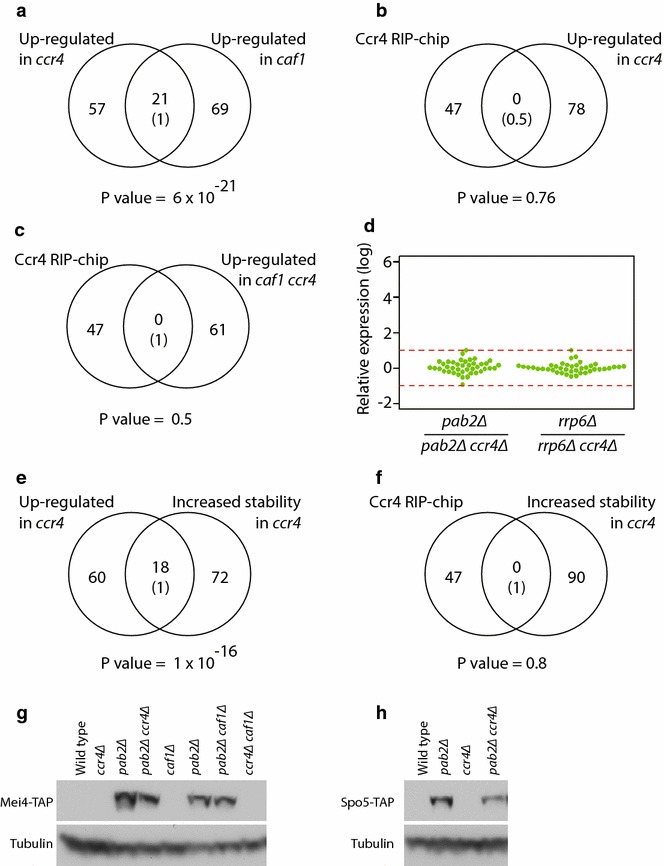
Ccr4-Not does not regulate turnover or translation of Mmi1 RNA targets. All comparisons presented are based on microarray data. **a** Venn diagrams comparing genes up-regulated in *ccr4* and *caf1* mutants relative to wild-type cells. The *numbers* in *parentheses* indicate the expected overlap if randomly generated lists of the corresponding sizes were used. The *p* value of the observed overlap is displayed under the diagrams. **b** As in A, comparing mRNAs associated with Ccr4 and those induced in *ccr4* mutants. **c** As in (**a**), comparing mRNAs that interact with Ccr4 and those up-regulated in *caf1 ccr4* double mutants. **d** Comparison of expression levels of Ccr4-associated mRNAs between *pab2* and *ccr4 pab2* mutants (*left*), and between *rrp6* and *rrp6 pab2* mutants. Each mRNA is represented by a *dot*. The *lines* correspond to a twofold difference between the samples. **e** As in (**a**), comparing mRNAs up-regulated in *ccr4* mutant and those displaying increased stability in *ccr4*. **f** As in (**a**), comparing mRNAs associated with Ccr4 and those stabilized in *ccr4* mutants. **g** Cell extracts were prepared from the indicated strains, all of which contained TAP-tagged Mei4. Samples were probed with antibodies against TAP (*top*) or tubulin (*bottom*). The interaction between TAP-Mmi1 and Red1-myc was used as a positive control. **h** As in (**g**), using strains expressing Spo5-TAP.

A major function of the Ccr4-Not complex is the shortening of poly(A) tails through its deadenylase activity, which often results in mRNA destabilization. To explore whether Ccr4 regulates the stability of Mmi1 targets, we used DNA microarrays to measure mRNA levels in mutants defective for either deadenylase catalytic subunit (*ccr4* and *caf1*). Both mutants grew slowly compared to wild-type cells, mated with low efficiency, and displayed similar changes in the levels of 78–90 RNAs (Fig. 3a; Additional file 1: Table S2). However, the relative levels of most Ccr4-bound RNAs were not affected in any of the mutants (Fig. 3a, b). The only exception to this was *mei4*, which showed a small but significant increase of 1.8-fold in *caf1* mutants, and a rise of 1.4-fold in *ccr4Δ* cells (that did not pass the statistical significance threshold, see “Methods”). We examined the possibility that Ccr4 and Caf1 act redundantly by constructing a *caf1Δ ccr4Δ* double mutant. The double mutant was viable and displayed changes in gene expression similar to those of the single mutants (Additional file 1: Table S2). Notably, none of the Ccr4-bound mRNAs showed significant changes in gene expression (Fig. 3c). Again, the *mei4* mRNA was mildly overexpressed (1.6-fold), but the change did not pass the significance threshold. These data indicate that Caf1 and Ccr4 do not regulate the levels of the early meiotic mRNAs with which they associate in a stable manner.

We then considered the possibility that the Ccr4-Not complex is part of a back-up system that degrades those mRNAs that escape the Mmi1/exosome system. As this mechanism is highly efficient (the levels of Mmi1 targets are very low in vegetative cells [25]), the role of Ccr4-Not might be masked in wild-type cells. By contrast, if the Mmi1 pathway were partially compromised, Ccr4-Not should become more important and its inactivation would lead to increased levels of Mmi1 targets. To test this model, we used the *pab2* gene, which encodes a nuclear poly(A) binding protein [46], and *rrp6*, which encodes a nuclear-specific catalytic subunit of the exosome [47]. Pab2 and Rrp6 cooperate with Mmi1, and Mmi1 targets are overexpressed in *pab2* and *rrp6* mutants [33–35]. We made double mutants of *pab2* and *ccr4*, and of *rrp6* and *ccr4*, and compared their expression profiles to those of the *pab2* and *rrp6* single mutants, respectively. Most Ccr4/Mmi1 target levels were not increased in the double mutants (Fig. 3d). This result is inconsistent with Ccr4-Not functioning as a back-up mechanism to the Mmi1 RNA degradation system.

In budding yeast, mutations in the *CCR4* and *POP2/CAF1* genes cause opposite changes in mRNA stability and transcription for certain mRNAs, and thus do not lead to large changes in steady-state mRNA levels [48]. To investigate whether this phenomenon occurs in fission yeast, we measured genome-wide decay rates in wild-type and *ccr4* mutants [26]. While mRNAs stabilized in the mutant correlated with those overexpressed (Fig. 3e), the decay rates of the Mmi1 targets (including *mei4*) were not affected (Fig. 3f). This is in stark contrast to *pab2* and *red1* mutants, in which Mmi1 targets are stabilized [34]. Altogether, these data indicate that a subset of mRNAs is destabilized by Ccr4-Not but does not interact stably with the complex (and does not include Mmi1 targets). By contrast, Mmi1 targets are physically associated with Ccr4-Not, but their stability is not regulated by Ccr4-Not.

Ccr4-Not regulates the translation of specific mRNAs in multicellular eukaryotes [3, 4]. To examine this possibility, we tagged the proteins encoded by two Ccr4/Mmi1 targets (Mei4 and Spo5) with a tandem affinity purification (TAP) epitope. We used a tagging strategy that preserved the integrity of both 5′ and 3′ UTRs to maintain the endogenous transcriptional and posttranscriptional regulation of the targets (see “Methods”). Mei4 protein was expressed at very low levels and could not be detected by Western blot, neither in wild-type cells nor in single or double mutants of *caf1* and *ccr4* (Fig. 3g). By contrast, it was clearly detectable in mutants in which their mRNA levels were induced (*pab2*). Moreover, deletion of *caf1* or *ccr4* in a *pab2* mutant background did not lead to a further increase in Mei4 protein levels (Fig. 3g). Similarly, Spo5 levels were not increased by deletion of *ccr4* in neither wild-type nor *pab2Δ* cells (Fig. 3h). These results suggest that Ccr4-Not does not regulate (or has a minimal impact on) the translation of its meiotic targets.

### Ccr4-Not is required for heterochromatin integrity in genomic island and subtelomeres

**Fig. 4:**
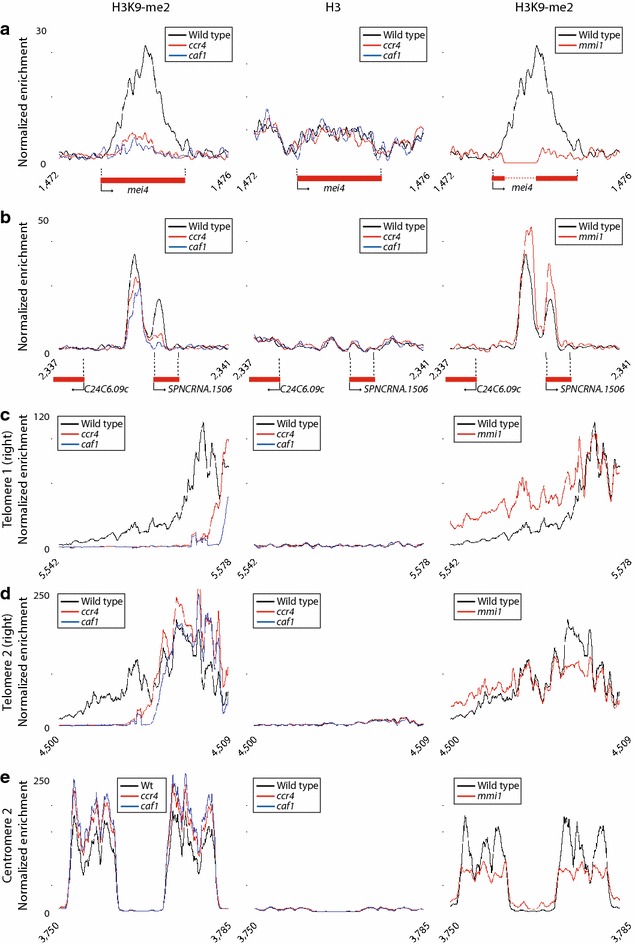
Caf1 and Ccr4 are required for heterochromatin integrity in islands and subtelomeric regions. Enrichment in H3 or H3K9-me2 in heterochromatin islands determined using ChIP-seq. The y axes show the normalized enrichment in the indicated immunoprecipitate and the x axes correspond to the position along the chromosome. Data are shown for wild-type cells and for the indicated mutants. The *mei4* gene is deleted in the *mmi1Δ* strain (the deleted region is indicated by a *dashed line*). **a** *mei4* locus. **b** *SPNCRNA.1506* region. **c** Right subtelomere of chromosome 1. **d** Right subtelomere of chromosome 2. **e** Centromere of chromosome 2.

Mmi1 is also required for the maintenance of heterochromatin in genomic ‘islands’, some of which correspond to Mmi1 target genes. This function is carried out, at least partially, by targeting Red1 and components of the RNAi complex RITS to specific genes [37, 38]. However, inactivation of components of the RITS complex or heterochromatin causes no or only moderate changes in the levels of *mmi1* targets [37–39]. To investigate whether Ccr4-Not is involved in this role of Mmi1, we applied chromatin immuno precipitation analysed by sequencing (ChIP-seq) for genome-wide profiling of histone H3K9 di-methylation (H3K9-me2, a marker of heterochromatin) in *ccr4Δ*, *caf1Δ* and wild-type cells. As a control for histone occupancy, the levels of histone H3 were measured in parallel and used to normalize the enrichment in H3K9 methylation (Additional file 1: Table S3). We detected reproducible enrichments above background in only 6 of the 21 originally reported heterochromatin islands (Additional file 1: Table S4) [38]. This discrepancy might reflect technical issues, as the previous study used tiling microarrays that provide less resolution and tend to have higher noise levels. The detected islands included four clear targets of Mmi1 (*mcp7*, *ssm4*, *moa1* and *mei4*), as well as two ncRNAs (*SPNCRNA.1506* and *SPNCRNA.394*). Five of the islands displayed clear and reproducible decreases in H3K9-me2 in both *ccr4* and *caf1* mutants, suggesting that the structure of heterochromatin in these loci is compromised (Additional file 1: Table S4; Fig. 4a, b, Additional file 2: Figure S1, Additional file 3: Figure S2). The extent of the reductions was varied, with the island containing *mei4* showing the strongest effects (Additional file 1: Table S4; Fig. 4a). The island that includes *SPNCRNA.1506* contained two blocks of heterochromatin, one of which appeared to be much more sensitive to mutations in *caf1* and *ccr4* (Fig. 4b). Mutants in *mmi1* showed reduced levels in H3K9-me2 in the four loci corresponding to Mmi1 targets (Additional file 1: Table S4; Fig. 4a, Additional file 3: Figure S2A, B) but not in the two ncRNAs (Additional file 1: Table S4; Fig. 4b and [38]), indicating that Ccr4-Not can regulate heterochromatin independently of Mmi1.

The *ccr4Δ* and *caf1Δ* mutants also led to a dramatic reduction in H3K9-me2 levels (but not in histone occupancy) in subtelomeric regions (Additional file 1: Table S5; Fig. 4c, d, Additional file 3: Figure S2C, D). The most distal part of the subtelomeric region appeared unaffected, including the *tlh1* and *tlh2* genes. These genes contain sequences homologous to those of centromeric repeats, and are capable of nucleating heterochromatin [49]. The effect of the mutations was specific to the telomeres, as other major heterochromatic regions (mating type locus and centromeres) were unaffected (Additional file 1: Tables S6, S7; Fig. 4e). Moreover, *ccr4* and *caf1* mutations did not cause decreased methylation in HOODs, including Tf2 transposons (Additional file 1: Tables S8, S9). Given these results, we also investigated whether Mmi1 is required for heterochromatin integrity at subtelomeric regions. Although H3K9-me2 relative amounts were decreased in genomic islands, its levels were not reduced (and appeared even increased, Fig. 4c, Additional file 3: Figure S2D) in subtelomeric regions (Additional file 1: Table S5). This finding suggests that Ccr4 regulates heterochromatin integrity in genomic islands in a Mmi1-dependent fashion, while the same role at subtelomeres is mediated by a different, yet unidentified factor.

**Fig. 5:**
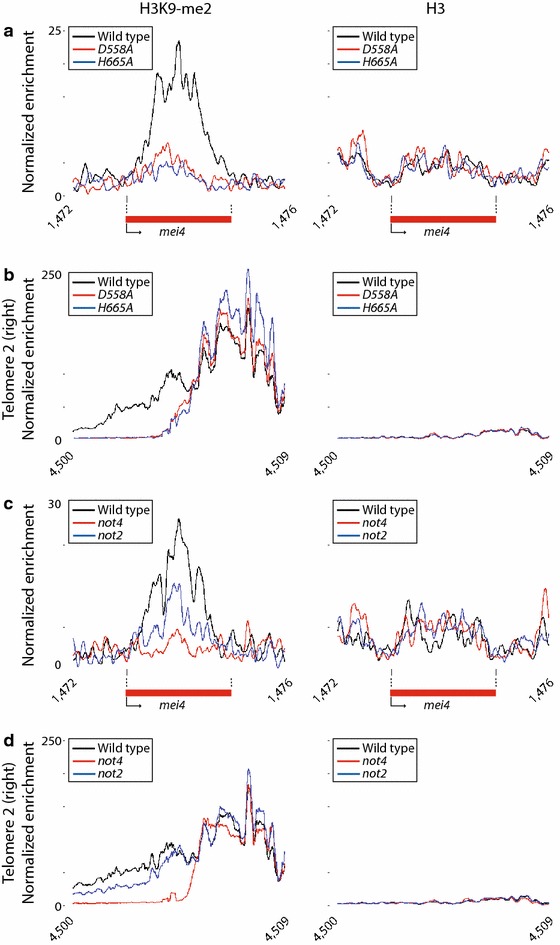
Effect of *ccr4* catalytic mutations, and of *not2* and *mot2*/*not4* inactivation, on heterochromatin integrity of islands and subtelomeric regions. Enrichment in H3 or H3K9-me2 in heterochromatin islands determined using ChIP-seq. The y axes show the normalized enrichment in the indicated immunoprecipitate and the x axes correspond to the position along the chromosome. Data are shown for wild-type cells and for the indicated mutants. **a** *mei4* locus in wild-type, *ccr4*-*D558A* and *ccr4*-*H665A*. **b** Right subtelomere of chromosome 2 in wild-type, *ccr4*-*D558A* and *ccr4*-*H665A*. **c** *mei4* locus in wild-type, *not2Δ* and *mot2*/*not4Δ*. **d** Right subtelomere of chromosome 2 in wild-type, *not2Δ* and *mot2*/*not4Δ.*

We next investigated whether the deadenylase activity of Ccr4-Not is important for its role in heterochromatin formation. We generated two independent point mutants in key residues of the catalytic site of Ccr4 (D558A and H665A) [50]. Similar to cells carrying a deletion in *ccr4*, both mutants grew slowly and displayed low efficiency of mating. In addition, their microarray profiles overlapped with that of *ccr4Δ* (Additional file 4: Figure S3). ChIP-seq for histone H3K9-me2 revealed a pattern almost identical to those of *ccr4Δ* and *caf1Δ*, including a decrease in several heterochromatin islands (*mei4*, *mcp7* and *SPNCRNA.1506*) and a prominent reduction in subtelomeric regions (Additional file 1: Tables S4–S9; Fig. 5a, b). These results suggest that the deadenylase catalytic activity of the complex is essential for its regulation of heterochromatin.

We then explored if other subunits of the complex are necessary for heterochromatin regulation. We used ChIP-seq to monitor H3 and H3K9-me2 levels in mutants in *mot2*/*not4* (which encodes a ubiquitin ligase) and *not2* (encoding a core component of the complex with no known enzymatic activity). Mutation of *not2* caused a decrease in H3K9-me2 in the *mei4* and *mcp7* islands, but did not affect heterochromatin in subtelomeric regions (Additional file 1: Tables S4–S5; Fig. 5c, d). By contrast, *mot2*/*not4* mutants displayed a phenotype similar to those of *caf1* and *ccr4*, with a striking reduction in H3K9-me2 in some islands (Additional file 1: Table S4; Fig. 5c) and in all subtelomeres (Additional file 1: Table S5; Fig. 5d). Similar to *caf1* and *ccr4* mutants, the effect was also highly specific, and transposons, centromeres and HOODs were not affected (Additional file 1: Tables S6, S8–S9).

### Ccr4 interacts with the chromodomain protein Chp1

**Fig. 6:**
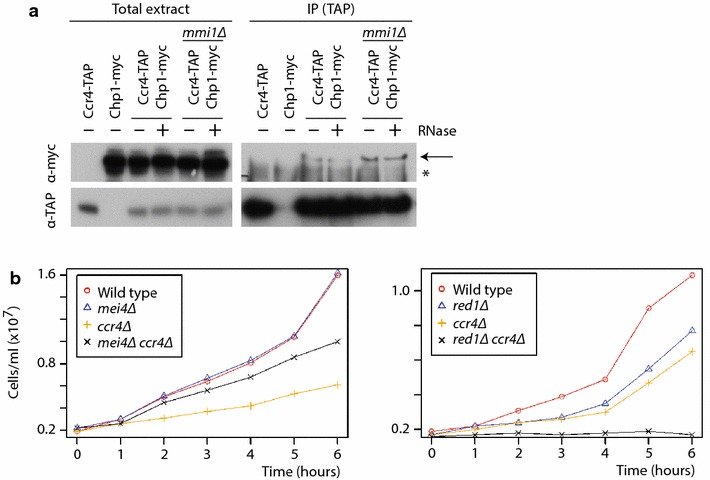
Physical and genetic interactions of Ccr4. **a** Cell extracts were prepared from the indicated strains and mock-treated (−) or treated with RNase (+). Immunoprecipitations were carried using antibodies against TAP. Samples were probed with antibodies against myc (*top*) or TAP (*bottom*). *Arrow* the position of the specific band corresponding to Chp1-myc, *star* a non-specific band. **b** *Left*: *ccr4Δ* growth defect is partially suppressed by deletion of *mei4*. Cells of the indicated genotypes were grown in YE medium and the cell number estimated from the optical density of the culture. *Right*: *ccr4* mutants show synthetic negative interactions with *red1* mutants.

If Ccr4-Not is involved in heterochromatin formation and/or maintenance, it would be expected to interact with other proteins important for this process. Indeed, Ccr4 coprecipitated with the chromodomain protein Chp1 in an RNA-independent manner. Moreover, the Ccr4-Chp1 interaction did not require Mmi1, in agreement with our results that Ccr4 is required for heterochromatin integrity in both Mmi1-dependent and Mmi1-independent pathways (Fig. 6a). We then used ChIP-seq to monitor the distribution of Chp1 on heterochromatin in wild-type and *ccr4* mutants (Additional file 1: Table S10). In wild-type cells, Chp1-TAP was enriched in a few heterochromatin islands (*mcp7*, *mei4* and *SPNCRNA.1506*) (Additional file 1: Table S11), subtelomeric regions (Additional file 1: Table S12) and centromeres (Additional file 1: Table S13). Consistent with our previous results, Chp1-TAP levels were reduced in *ccr4* mutants on islands and subtelomeres, but not on centromeres (Additional file 1: Tables S11–S13). Thus, Ccr4-Not is required for normal accumulation of Chp1 in both Mmi1-dependent and Mmi1-independent heterochromatin.

### Genetic interactions between *ccr4* and components of the Mmi1 degradation pathway

Finally, we tested whether the regulation of Mmi1 targets by Ccr4 has functional significance. The *mei4* gene is the main target of the Mmi1 system, and inactivation of Mei4 is sufficient to rescue the lethality of *mmi1* mutants. Similarly, we found that deletion of *mei4* strongly improved the growth of *ccr4Δ* cells, although the growth rate of the double mutant did not reach that of wild-type cells (Fig. 6b). This lack of complete rescue may be due to Mmi1-independent functions of the Ccr4-Not complex. Moreover, *ccr4* mutations showed strong negative interactions with mutations in *red1* and *rrp6* (Fig. 6b). This finding suggests that Ccr4 acts in a different pathway than Red1 and Rrp6, but all three proteins cooperate in the silencing of Mmi1 targets.

## Discussion

We report the surprising finding that Ccr4-Not is required for the integrity of heterochromatin signatures at islands and subtelomeric regions. As Ccr4-Not interacts with the Mmi1 protein and its RNA targets, it is likely that Mmi1 recruits the complex to the vicinity of heterochromatic islands using a similar mechanism to that used for the RITS complex. By contrast, Ccr4-Not does not copurify with RNAs from subtelomeric genes (Additional file 1: Table S1), and Mmi1, Red1 and Rrp6 are not required for the integrity of subtelomeric heterochromatin (Additional file 1: Table S5; Fig. 4c, d, Additional file 3: Figure S2C, D) and [18]. This suggests that Ccr4-Not is targeted to subtelomeric genes using a different pathway that does not involve Mmi1 and stable association with subtelomeric RNAs. The specificity of the Not2 phenotype for heterochromatins islands may indicate a role in the recruitment of Ccr4-Not to Mmi1 targets. The lack of effect of mutations in *ccr4* and *caf1* on the degradation and translation of Mmi1 targets are consistent with the nuclear localization of the Mmi1 protein [27].

If Ccr4-Not had a direct function in heterochromatin assembly or maintenance, it would be expected to be associated with heterochromatic loci and proteins involved in heterochromatin formation. Consistently, Ccr4 associates with Chp1 in a Mmi1-independent manner. We tried mapping the location of Ccr4 using ChIP-seq (CC and JM, unpublished data), but were unable to detect specific enrichments. This negative result may indicate that the association of Ccr4-Not with chromatin is transient and/or indirect.

Telomeric heterochromatin is nucleated by two independent pathways, and later spreads towards the inside of the chromosome. The first nucleation mechanism is mediated by the telomere-binding protein Taz1, while the second one relies on RNAi and small regions of homology to centromeric repeats [49, 51]. H3K9-me2 enrichment at the most distal parts of the subtelomeres was unaffected in *ccr4*, *caf1* and *mot2*/*not4* mutants, suggesting that Ccr4-Not is dispensable for heterochromatin nucleation. Mutations in *swi6*, which is essential for heterochromatin spreading [52], show a similar H3K9-me2 pattern as do mutations in *ccr4, caf1,* and *mot2*/*not4* (although H3K9-me2 in distal parts of the telomere is partially reduced in *swi6* but not at all in *ccr4*, and *mot2*/*not4* mutants) [17, 49]. These results might indicate that Ccr4-Not is necessary for heterochromatin spreading, thus determining the location of the boundary between euchromatin and heterochromatin. In the case of heterochromatin islands, a small accumulation of H3H9-me2 is present in the mutants, suggesting that the machinery that initiates heterochromatin formation is still partially functional.

How does the Ccr4-Not complex participate in heterochromatin formation or maintenance? The three proteins of the complex with known catalytic activities (the deadenylases Caf1 and Ccr4, and the ubiquitin ligase Mot2/Not4) are essential for heterochromatin integrity at both islands and subtelomeres. By contrast, the non-catalytic subunit Not2 has an effect on the islands, but not on subtelomeric regions. In the cytoplasm, the deadenylase activity of Ccr4-Not leads to shortening of poly(A) tails [1]. Ccr4 is the major deadenylase enzyme in vivo in budding yeast, whereas Caf1 anchors Ccr4 to the complex [50, 53]. We show that two independent single-amino acid mutants in the catalytic site of Ccr4 phenocopy a deletion of *ccr4*, strongly suggesting that the deadenylase function of Ccr4 is required for heterochromatin formation. The Mmi1-mediated RNA degradation pathway involves the addition of a poly(A) tail to Mmi1 RNA targets, which is required for degradation by the nuclear exosome. Indeed, mutations in *pab2* and *rrp6* cause the accumulation of Mmi1 targets containing abnormally long poly(A) tails [33]. Ccr4-Not might counteract the polyadenylation of Mmi1 targets, possibly increasing their stability and thus allowing them to participate in heterochromatin formation before being targeted for degradation. However, we did not observe clear changes in the length of the poly(A) tail of the *mcp7* RNA in *ccr4* mutants (CC and JM, unpublished data). It is possible that Mmi1 targets are degraded as soon as the poly(A) length increases, and that the steady-state poly(A) length is thus not affected.

The Not4 ubiquitin ligase of the complex has been implicated in protein quality control in the cytoplasm [54]. Substrates on Not4 include the small ribosomal protein Rps7A [55]. Ubiquitination of Rsp7 does not lead to protein degradation, but is essential for cell viability. Not4 has also been implicated in the regulation of a histone modification, the trimethylation of histone H3 at lysine 4 (H3K4-me3). This effect is mediated through Not4-mediated ubiquitination of the Jhd2 demethylase, which targets it to the proteasome [56]. Ubiquitination is also involved in the establishment of heterochromatin boundaries in fission yeast. The Cul4-Ddb1-Cdt2 ubiquitin ligase down-regulates the levels of the silencing inhibitor protein Epe1. In *ddb1* mutants, Epe1 spreads from the boundaries into the body of heterochromatin regions [21]. However, *epe1Δ* mutants do not seem to display clear changes in the boundary of the heterochromatin block of the telomere examined [21]. Other histones, such as H2B, are also regulated by ubiquitination [57, 58]. Further work will be required to identify the targets of Mot2/Not4, and to ascertain if their ubiquitination regulates their function or triggers their proteolysis.

Although the effects of *ccr4* and *caf1* mutations on heterochromatin around the *mei4* locus are strong, they lead to only minor increases in *mei4* mRNA levels. By contrast, mutations in genes that regulate the stability of Mmi1 targets (such as *pab2* or *red1*) cause a major accumulation of *mei4* and other Mmi1 targets [33–37]. This finding is consistent with the Mmi1 RNA elimination system being highly efficient. However, double mutants in *ccr4* and *pab2* did not display any further increase in *mei4* levels, suggesting that the *mei4* locus is only partially derepressed in *caf1Δ* and *ccr4Δ* cells, possibly because of the residual heterochromatin present in the mutant (Fig. 4a). Notably, this small level of derepression has clear functional consequences, as evidenced by the rescue of *ccr4* mutations upon deletion of *mei4* (Fig. 6b). The *caf1Δ* and *ccr4Δ* cells also did not show any evident increase in RNA levels of genes located in subtelomeric regions, many of which are up-regulated during nitrogen starvation [25]. It is possible that expression of these genes requires the removal of a repressing factor (heterochromatin) as well as the presence of an additional activating signal, such as a transcription factor.

## Conclusions

The Ccr4-Not complex regulates gene expression at multiple levels, both transcriptional and posttranscriptional. We have identified a novel role of this multiprotein complex in maintaining heterochromatin integrity at subtelomeres and heterochromatin islands. Surprisingly, all catalytic subunits of Ccr4-Not are required for this function. In heterochromatin islands, Ccr4-Not is targeted to the corresponding mRNAs by the YTH-family protein Mmi1. Another YTH protein from *S. cerevisiae* (Pho92) coprecipitates with Caf1/Pop2 [59], suggesting that the recruitment of Ccr4-Not to mRNAs by YTH proteins may be widespread. Given the conservation of the Ccr4-Not complex, it is likely that this role will be relevant in higher eukaryotes.

## Methods

### Fission yeast methods

Standard methods and media were employed [60]. For Mei4 TAP tagging, a 550 base pair fragment corresponding to the 3′ end of the *mei4* coding sequence was generated by PCR and cloned upstream of TAP into the SalI/PacI sites of pFA6a-4X-TAP. A 730 base pair fragment from the 3′ untranslated region was obtained in a similar way and inserted downstream of TAP, using the AscI and BglII restriction sites. The plasmid was linearized using MfeI (which cuts in the coding sequence) and transformed into fission yeast [61]. For Spo5 TAP tagging, the *adh1* terminator of pFA6aTAP was replaced with a 100 base pair fragment corresponding to the *spo5* 3′ untranslated region. This plasmid was targeted to the *spo5* locus by homologous recombination using flanking regions cloned into the plasmid. Mmi1 was TAP-tagged at the N-terminus using a Cre-loxP method as described [62]. Other taggings and gene deletions were carried out using standard PCR-based methods [61, 63]. A conserved aspartic acid residue (position 558) or a histidine (position 665) in the Ccr4 protein was mutated to alanine. Both amino acids are part of the catalytic site and essential for its activity [50]. The mutations were introduced into the endogenous *ccr4* locus were generated using a two-step strategy [64]. First, 500 base pairs of the 3′ end of the *ccr4* gene were deleted in an *ura5*-*14 lys7*-*2* strain with a cassette containing both the *ura5* and the *lys7* genes. The cassette was then replaced by homologous recombination with a linear fragment of 500 base pairs of the 3′end gene of the *ccr4* gene containing either of the above mutations. The DNA fragments containing the mutations were synthesized by GeneArt Gene Synthesis service (Life Technologies, USA). Uracil auxotrophic clones were selected by resistance to 5-fluoroorotic acid (5-FOA), and correct recombination was confirmed by the loss of the *lys7* gene and by genomic sequencing. For transcriptome analysis, RIP-chip, and ChIP-seq experiments, cells were grown in yeast extract media (YE) at 32°C. For the temperature-sensitive mutant *rrp6*-*32*, cells were grown on YE at 25°C and incubated at 36°C for 2 h before collection. Additional file 1: Table S14 lists all the strains used in this work.

### Preparation of cell extracts, immunoprecipitation and protein detection

RIP-chip experiments were performed exactly as described [34]. For coimmunoprecipitation experiments, whole cell extracts were prepared from 100 ml of cells grown in YE at 32°C to a cell density of 8 × 10^6^ cells/ml. Cells were resuspended in lysis buffer [20 mM Tris HCl, 140 mM KCl, 1.8 mM MgCl_2_, Np-40 0.1%, 1:100 protease inhibitor cocktail (Sigma P8340) and 1 mM PMSF], and lysed using a Fastprep 24 bead-beater (one cycle of 13 s at level 6). RNase treatment was performed by incubating 300 µl of extract with 15 μl of RNase cocktail (Life Technologies AM2286) for 30 min at room temperature. The efficiency of the RNase treatment was assessed by agarose gel electrophoresis. Cell lysates were then incubated with 100 μl of magnetic beads (Pan Mouse IgG, Life Technologies) coated with anti-protein A antibody (clone SPA-27, Sigma) for 2 h at 4°C. The beads were washed 5 times in 0.5 ml of lysis buffer containing 10% glycerol and 0.2 mg/ml heparin, and the immunoprecipitates were resuspended in lysis buffer. TAP-tagged proteins were detected by Western blot using peroxidase-antiperoxidase complex (Sigma), myc-tagged proteins with the 9E10 monoclonal antibody (Abcam), FLAG-tagged proteins with the M2 monoclonal (Sigma), and tubulin with the B-5-1-2 monoclonal (Sigma).

### Determination of mRNA levels and stabilities using microarrays

Total RNA was purified using hot phenol extraction [65]. mRNA decay rates were determined using in vivo labelling with 4-thiouridine as described [34], using a labelling time of 7 min.

### Microarray protocols and analysis

For expression analysis and RNA stability analysis, fluorescently labelled probes were prepared exactly as described [34]. Labelled cDNAs were hybridized to custom-designed oligonucleotide microarrays manufactured by Agilent [45]. Microarrays were scanned with a GenePix 4000A microarray scanner and analysed with GenePix Pro 5.0 (Molecular Devices). Microarray data for transcriptome analysis were normalized using Loess, and for RNA stability determination expression ratios were median-centred. Differentially expressed genes were defined using Significance Analysis of Microarrays with a false discovery rate smaller than 0.005 [66]. The analysis of RIP-chip experiments was performed as follows: relative enrichments for all detected RNAs (immunoprecipitated versus total RNA) were log-transformed and mean-centred. A z-score was then calculated, and mRNAs whose z-score was above 2.5 in both biological replicates (for experiments performed twice) or two out of three (for experiments carried out three times) were selected. For all experiments, the significance of the overlap between gene sets was determined using Fisher’s exact test.

### ChIP-seq experiments

200 ml of cells were grown in YE at 32°C to a density of 1 × 10^7^ cells/ml. Cells were fixed by the addition of formaldehyde to a final concentration of 1%, followed by incubation for 30 min at 32°C. The reaction was stopped with 10 ml of 2.5 M glycine. Cells were washed extensively in phosphate-buffered saline solution (PBS), resuspended in lysis buffer [50 mM HEPES pH 7.6, 140 mM NaCl, 1 mM EDTA, 0.1% Triton X-100, 1 mM PMSF, and 1× complete protease inhibitor cocktail (Roche)], and lysed with a Fastprep 24 bead-beater (five cycles of 20 s at level 6.5). Chromatin was sheared by sonication with a Bioruptor (Diagenode; six cycles of 5 min with 30 s on/30 s off at high intensity). The lysates were cleared by centrifugation at 20,000*g* for 10 min, and adjusted to 500 μl at 6 μg/μl total protein, as determined by the BCA assay. 100 µl of magnetic beads (Pan Mouse IgG or protein G, Life Technologies) was blocked with bovine serum albumin (0.5% w/v BSA), and coated with 5 µg of mouse monoclonal antibodies against methylated H3K9 (H3K9me2, Abcam ab1120) or with anti-histone H3 rabbit polyclonal antibodies (Abcam ab1791), respectively. For Chp1-TAP ChIP-seq, 100 µl of magnetic beads coated with mouse IgG was used. The extracts were incubated overnight with the coated beads at 4°C with rotation. Next, the beads were washed twice with 0.8 ml of 50 mM HEPES pH 7.6, 150 mM NaCl, 1 mM EDTA, 1% Triton X-100, and 0.1% sodium deoxycholate, twice in the same buffer containing 500 mM NaCl, twice in 10 mM Tris–HCl pH 8, 250 mM LiCl, 1 mM EDTA, 0.5% NP-40, and 0.5% sodium deoxycholate, and once in Tris–EDTA solution. Elution and crosslinking reversal were performed by incubating the beads in 200 μl of 10 mM Tris–HCl pH 8, 1 mM EDTA, and 1% SDS overnight at 65°C. DNA was purified with the MinElute PCR purification kit (Qiagen, The Netherlands) following the manufacturer’s protocol, and eluted in 10 μl. Libraries were prepared using the Next ChIP-seq Library Prep Master Mix Set for Illumina (New England Biolabs) according to the manufacturer’s instructions, and sequenced using an Illumina MiSeq system.

### Quantification of ChIP by qPCR

ChIP was performed as described above, and samples diluted 1:400 before quantification. Quantitative analysis of input DNA and immunoprecipitated DNA levels was performed using Sybr Green JumpStart Taq ReadyMix (Sigma) in a real-time PCR machine (Rotor-Q Gene, Quiagen) using the following program: 10 min at 95°C; 40 cycles of 95°C for 10 s, 60°C for 15 s and 72°C for 30 s, with each cycle followed by a 5-second melting ramp of 1°C steps (from 72 to 95°C) for acquisition. The following primers were used: qPCR_mei4_DSR_F (CTTCAAATGTTGCTGCCGAAG), qPCR_mei4_DSR_R (GAGTTTCAGCATTTGGTTTAGG), cdc2.2_F (CCACTGGGGTTGATATTTGG) and cdc2.2_R (CGTTTCCAACGAGGAAATGT). Quantification of relative levels was performed as follows:

$${\text{ratio}}\;(mei{\it 4}/cdc{\it 2}) = 2^{{ - \left( {{\text{Ct}}:mei{\it 4}:{\text{IP}} - {\text{Ct}}:cdc{\it2}:{\text{IP}}} \right) - ({\text{Ct}}:mei{\it 4}:{\text{INPUT}} - {\text{Ct}}:cdc{\it 2}:{\text{INPUT}})}} \;{\text{or}}\;2^{{ - \Delta \Delta {\text{Ct}}}}$$

where Ct:*mei4* and Ct:*cdc2* correspond to the critical cycles.

### Sequencing analysis

All data pre-processing was performed with custom scripts written in Perl (http://www.perl.org*)* and all downstream statistical analysis used R (http://www.r-project.org/). For all analyses, *S. pombe* annotations and sequences available from GeneDB (http://old.genedb.org/), now PomBase (http://www.pombase.org/), on May 9, 2011 were used [67]. Reads were trimmed to 50 base pairs and aligned to the *S. pombe* genome using Bowtie (allowing up to three mismatches in −v mode) [68]. Aligned data were visualized using the Integrated Genome Viewer [69]. For Additional file 1: Tables S3–S9 (normalized H3K9-me2 occupancy), the number of reads mapping to every defined genomic feature was quantified and normalized to reads per kilobase per million reads. The density of H3K9 methylation was then normalized to that of histone H3.

### Availability of supporting data

All microarray and sequencing data have been deposited in ArrayExpress with the following accession numbers: E-MTAB-3036, E-MTAB-3490 and E-MTAB-3705 (ChIP-seq experiments); E-MTAB-3057, E-MTAB-3058 and E-MTAB-3065 (RIP-chip experiments); E-MTAB-3059, E-MTAB-3060 and E-MTAB-3493 (expression analysis).

## Additional files

Additional file 1: Combines Additional Tables S1–S14. **Table S1.** RNAs identified in RIp-chip experiments.**Table S2.** Differentially expressed genes in mutant cells. **Table S3.** H3K9-me2 density normalised by H3 occupancy for all genes. **Table S4.** H3K9-me2 density normalised by H3 occupancy for heterochromatin islands.** Table S5.** H3K9-me2 density normalised by H3 occupancy for subtelomeric regions. **Table S6.** H3K9-me2 density normalised by H3 occupancy for centromeres. **Table S7.** H3K9-me2 density normalised by H3 occupancy for the mating type locus. **Table S8.** H3K9-me2 density normalised by H3 occupancy for Tf2 transposons. **Table S9.** H3K9-me2 density normalised by H3 occupancy for HOODs. **Table S10.** Normalized Chp1TAP enrichment for all genes. **Table S11.** Normalized Chp1TAP enrichment in heterochromatic islands. **Table S12.** Normalized Chp1TAP enrichment in telomeric regions. **Table S13.** Normalized Chp1TAP enrichment in centromeric regions. **Table S14.** Strains used in this work. Additional file 2:
**Figure S1.** Effect of *ccr4* mutation on H3K9-me2 enrichment analysed by ChIP-qPCR. The y axis shows the normalized enrichment in H3K9-me2 of the *mei4* locus relative to *cdc2* quantified using qPCR for wild type cells (left) and *ccr4Δ* mutants (right). Data are shown for three independent biological replicates (single dots) and the average is represented by the horizontal line. The significance of the difference between both strains was calculated using Student’s t-test. Additional file 3:
**Figure S2.** Caf1 and Ccr4 are required for integrity of heterochromatin islands and subtelomeric regions. Enrichment in H3 or H3K9-me2 in subtelomeric and centromeric regions. The y axes show the normalized enrichment in the indicated immunoprecipitate and the x axes correspond to the position along the chromosome. Data are shown for wild type cells or for the indicated mutants. Note that the beginning and end of the annotated sequences of chromosome 3 do not display H3K9 accumulation in the mutants (data not shown), as they are separated from the telomeres by rRNA repeats. **(A)** *ssm4* locus **(B)** *mcp7* locus. **(C)** Left subtelomere of chromosome 1. **(D)** Left subtelomere of chromosome 2. Additional file 4:
**Figure S3.** Microarray profiles of *ccr4* catalytic site mutants. Venn diagrams comparing the genes overexpressed in *ccr4Δ*, *ccr4-H665A* and *ccr4-D558A* mutants. The numbers in parentheses indicate the expected overlap if randomly generated lists of the corresponding sizes were used.

## Abbreviations

5-FOA: 5-fluoroorotic acid
Ccr4-Not: carbon catabolite repression 4-negative on TATA-less
ChIP: chromatin immunoprecipitation
DSR: determinant of selective removal
dsRNA: double-stranded RNA
H3K9me2: dimethylated histone H3 at lysine 9
HOODs: heterochromatin domains
miRNA: microRNA
ncRNA: non-coding RNA
RIP: RNA-binding protein Immunoprecipitation
RITS: RNAi-induced transcriptional silencing
RNase: Ribonuclease
siRNA: small interfering RNA
TAP: tandem affinity purification
TRAMP: Trf4/Air2/Mtr4 Polyadenylation
YE: yeast extract
YTH: YT521-B homology
