# Supplementary material for: Role of Ccr4-Not complex in heterochromatin formation at meiotic genes and subtelomeres in fission yeast
Source: Epigenetics Chromatin. 2015 Aug 15;8:28. doi: 10.1186/s13072-015-0018-4 (PMC4536793; doi:10.1186/s13072-015-0018-4)
Supplement: Additional file 2: — Figure S1. Effect of ccr4 mutation on H3K9-me2 enrichment analysed by ChIP-qPCR. The y axis shows the normalized enrichment in H3K9-me2 of the mei4 locus relative to cdc2 quantified using qPCR for wild type cells (left) and ccr4Δ mutants (right). Data are shown for three independent biological replicates (single dots) and the average is represented by the horizontal line. The significance of the difference between both strains was calculated using Student’s t-test. [file 13072_2015_18_MOESM2_ESM.pdf]

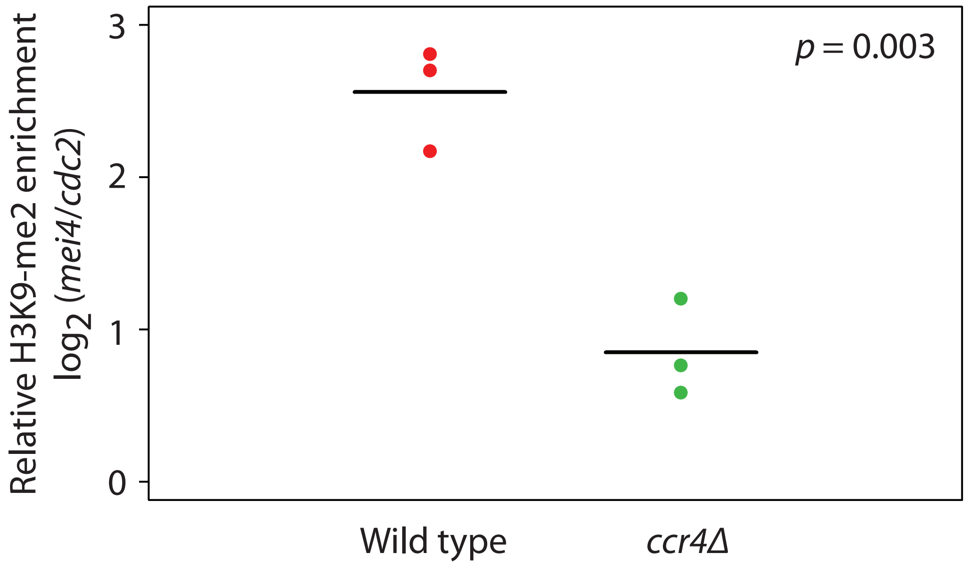

Figure S1. Effect of *ccr4* mutation on H3K9-me2 enrichment analysed by ChIP-qPCR.

The y axis shows the normalized enrichment in H3K9-me2 of the *mei4* locus relative to *cdc2* quantified using qPCR for wild type cells (left) and *ccr4Δ* mutants (right). Data are shown for three independent biological replicates (single dots, n=3) and the average is represented b[illegible] horizontal line. The significance of the difference between both strains was calculated using Student’s t-test.
