# Supplementary material for: Role of Ccr4-Not complex in heterochromatin formation at meiotic genes and subtelomeres in fission yeast
Source: Epigenetics Chromatin. 2015 Aug 15;8:28. doi: 10.1186/s13072-015-0018-4 (PMC4536793; doi:10.1186/s13072-015-0018-4)
Supplement: Additional file 3: — Figure S2. Caf1 and Ccr4 are required for integrity of heterochromatin islands and subtelomeric regions. Enrichment in H3 or H3K9-me2 in subtelomeric and centromeric regions. The y axes show the normalized enrichment in the indicated immunoprecipitate and the x axes correspond to the position along the chromosome. Data are shown for wild type cells or for the indicated mutants. Note that the beginning and end of the annotated sequences of chromosome 3 do not display H3K9 accumulation in the mutants (data not shown), as they are separated from the telomeres by rRNA repeats. (A) ssm4 locus (B) mcp7 locus. (C) Left subtelomere of chromosome 1. (D) Left subtelomere of chromosome 2. [file 13072_2015_18_MOESM3_ESM.pdf]

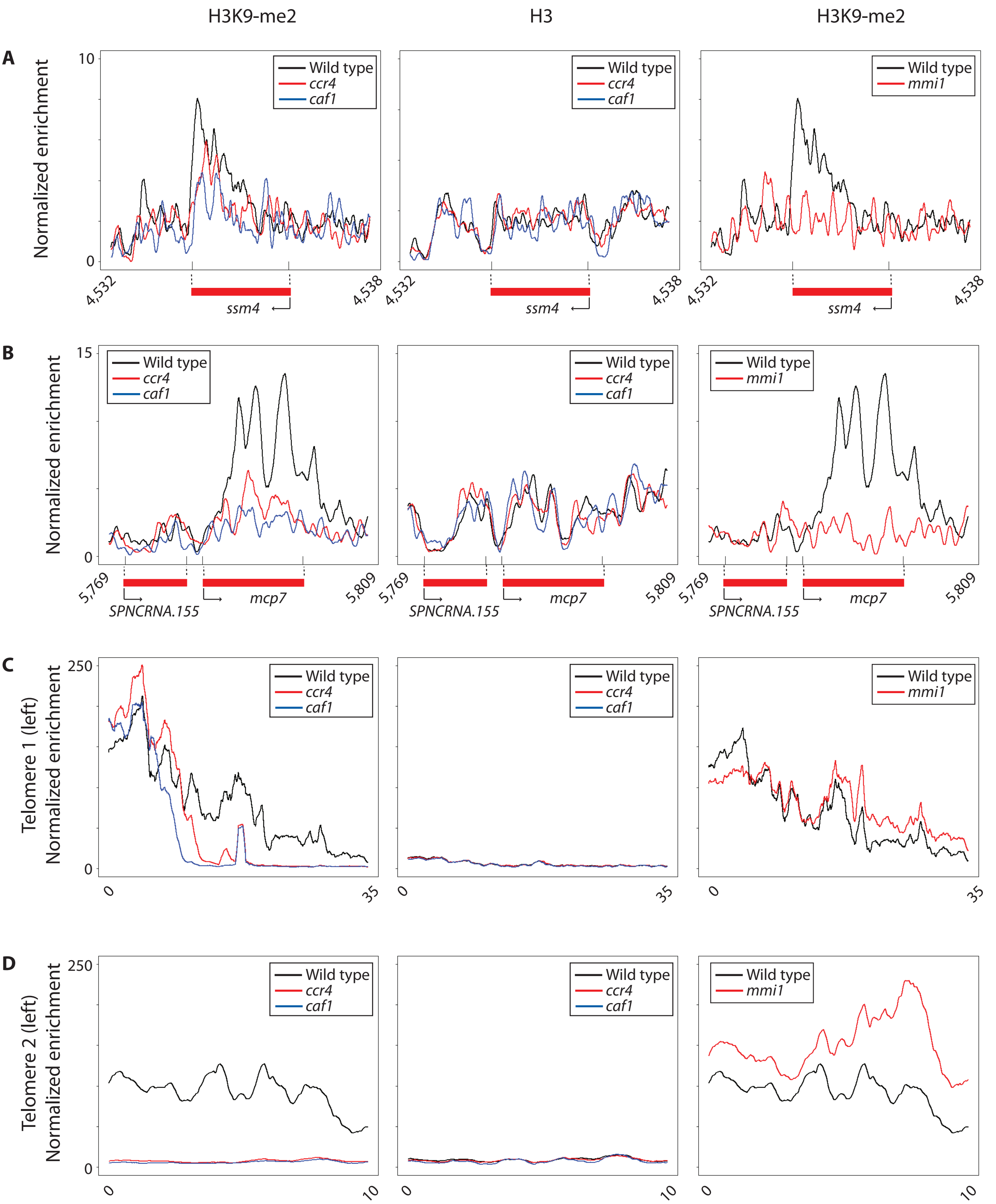

Figure S2. Caf1 and Ccr4 are required for integrity of heterochromatin islands and subtelomeric regions.

Enrichment in H3 or H3K9-me2 in subtelomeric and centromeric regions. The y axes show the normalized enrichment in the indicated immunoprecipitate and the x axes correspond to the position along the chromosome. Data are shown for wild type cells or for the indicated mutants. Note that the beginning and end of the annotated sequences of chromosome 3 do not display H3K9 accumulation in the mutants (data not shown), as they are separated from the telomeres by rRNA repeats. **(A)** *ssm4* locus **(B)** *mcp7* locus. **(C)** Left subtelomere of chromosome 1. **(D)** Left subtelomere of chromosome 2.
