# Supplementary material for: Role of Ccr4-Not complex in heterochromatin formation at meiotic genes and subtelomeres in fission yeast
Source: Epigenetics Chromatin. 2015 Aug 15;8:28. doi: 10.1186/s13072-015-0018-4 (PMC4536793; doi:10.1186/s13072-015-0018-4)
Supplement: Additional file 4: — Figure S3. Microarray profiles of ccr4 catalytic site mutants. Venn diagrams comparing the genes overexpressed in ccr4Δ, ccr4-H665A and ccr4-D558A mutants. The numbers in parentheses indicate the expected overlap if randomly generated lists of the corresponding sizes were used. [file 13072_2015_18_MOESM4_ESM.pdf]

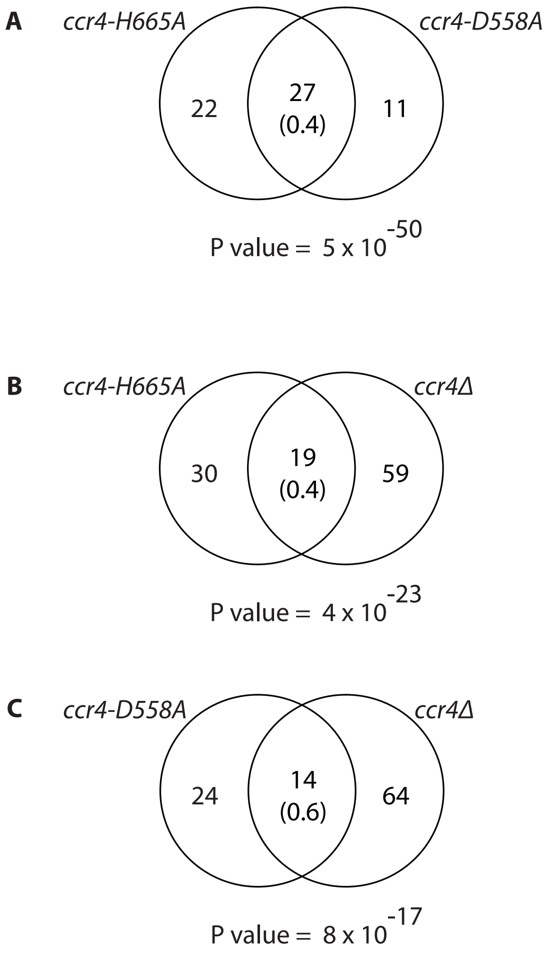

Figure S3. Microarray profiles of *ccr4* catalytic site mutants.

Venn diagrams comparing the genes overexpressed in *ccr4Δ*, *ccr4-H665A* and *ccr4-D558A* mutants. The numbers in parentheses indicate the expected overlap if randomly generated lists of the corresponding sizes were used.
